# Procalcitonin kinetics in critically ill patients: a retrospective analysis of the impact of renal dysfunction and renal replacement therapy

**DOI:** 10.1186/s44158-026-00405-8

**Published:** 2026-07-11

**Authors:** Francesco Cipulli, Beatrice Cazzetta, Marco Casarotti, Sara Miori, Eleonora Balzani, Adriano Anesi, Silvia De Rosa, Giacomo Bellani

**Affiliations:** 1 Anesthesia and Intensive Care 1, Santa Chiara Hospital, APSS, Largo Medaglie d’Oro 9, Trento, 38122 Italy; 2 Centre for Medical Sciences (CISMed), University of Trento, Via S. Maria Maddalena 1, Trento, 38122 Italy; 3 Department of Anesthesia and Intensive Care, University of Brescia, ASST Spedali Civili Di Brescia, Piazzale Spedali Civili 1, Brescia, 25123 Italy; 4 Department of Laboratory Medicine and Clinical Pathology, Santa Chiara Hospital, APSS, Largo Medaglie d’Oro 9, Trento, 38122 Italy

**Keywords:** Acute kidney injury, Biomarkers, Critical care, Intensive care unit, Procalcitonin, Renal replacement therapy, Sepsis, Septic shock

## Abstract

**Background:**

Despite its widespread use, several aspects of procalcitonin (PCT) kinetics remain unclear for guiding antibiotic therapy, particularly its behavior in acute kidney injury (AKI) and during renal replacement therapy (RRT). This study aimed to describe PCT kinetics in intensive care unit (ICU) patients with AKI, including those undergoing RRT, compared with patients without AKI.

**Methods:**

We conducted a single-center retrospective study including 396 ICU patients. At admission, PCT, C-reactive protein (CRP), and white blood cell (WBC) count were compared across AKI stages. During ICU stay, any subsequent rise in PCT > 2 µg/L was defined as a PCT peak-episode. PCTpeak was the highest value before a sustained decline, PCTvalley the following lowest value, and PCTdecay the average daily reduction from peak to valley. Group comparisons were conducted using ANOVA or Kruskal–Wallis tests, with Bonferroni correction as appropriate. Linear mixed-effects models were applied to account for repeated measures within patients.

**Results:**

CRP and WBC did not differ among AKI stages, whereas PCT increased progressively (*p* = 0.004). PCTpeak correlated with AKI severity (*p* = 0.001), while PCTdecay decreased progressively (*p* < 0.001). Among stage 3 patients, RRT was performed in 12.3% of cases and did not significantly affect daily PCT levels compared to non-RRT patients.

**Conclusion:**

AKI is associated with higher PCT peaks and reduced PCT decline, whereas RRT does not appear to significantly alter PCT kinetics.

**Supplementary Information:**

The online version contains supplementary material available at https://doi.org/10.1186/s44158-026-00405-8.

## Introduction

Sepsis and septic shock—affecting approximately 70% of intensive care unit (ICU) patients remain among the most frequent and challenging conditions encountered in the critical care field [1, 2]. Over the years, numerous biomarkers have been proposed both for identifying bacterial infections and for guiding antibiotic therapy in these life-threatening syndromes [3–5]. Among them, procalcitonin (PCT) has emerged as one of the most extensively studied and clinically adopted [6]. PCT is a polypeptide precursor of calcitonin, physiologically synthesized by the C-cells of the thyroid gland and involved in calcium homeostasis [7, 8]. During systemic bacterial infection, extra-thyroidal production of PCT is up-regulated by pro-inflammatory cytokines as tumor necrosis factor-α (TNF-α), Interleukin-1 (IL-1), and Interleukin-6 (IL-6) [9–12].

PCT has gained a prominent role in guiding antibiotic therapy discontinuation and de-escalation, helping to balance the need for effective treatment with the risk of overuse [13], with a sensitivity in detecting sepsis of 77% and specificity of 79% [14]. Over the past two decades, several trials—such as the PRORATA and ADAPT sepsis studies—have demonstrated that PCT-guided algorithms for initiating and discontinuing antibiotics significantly reduce antibiotic exposure without compromising patient survival, compared with standard care based on clinical judgment alone [15, 16].

However, procalcitonin (PCT) reliability can be compromised by several factors. False positives may occur in the context of major surgery, trauma, burns, or cardiac arrest, while false negatives are often observed in localized or early-stage infections [17, 18].

Despite its widespread clinical use, several aspects of PCT kinetics remain incompletely understood, particularly concerning its production and elimination pathways [19–21]. Among the unresolved questions, the influence of Acute Kidney Injury (AKI) and renal replacement therapy (RRT) on PCT decline [22–25], and thus on its plasma levels, is of special interest. Current evidence is limited and sometimes contradictory [26, 27]. Given the high prevalence of renal dysfunction among critically ill septic patients, understanding how AKI affects PCT kinetics is of great clinical relevance. If impaired renal function significantly reduced PCT decline, it could lead to a misinterpretation of the decay rate, potentially resulting in unnecessary prolongation of antibiotic therapy, exposing patients to adverse effects, and contributing to antimicrobial resistance. In this context, not only the absolute value of PCT may be relevant, but also its temporal evolution, including peak levels and subsequent decline; accordingly, the analysis of kinetic parameters may provide additional clinically meaningful information. Taking advantage of a large real-life dataset, this study aims to describe the kinetics of PCT in ICU patients without and with different degrees of renal failure, as defined by The Kidney Disease Improving Global Outcomes organization (KDIGO) classification and the possible impact of RRT on the circulating PCT levels.

## Methods

### Participants

This is a single-center, retrospective observational study conducted in the general ICU of Santa Chiara Hospital (Trento, Italy), enrolling patients admitted between June 2021 and April 2023. All adult patients admitted to the ICU were eligible for inclusion. Patients without any PCT_peak_ were excluded. Additional exclusion criteria were age < 18 years, metastatic cancer, pregnancy, and refusal to provide consent.

### Ethics

Although the study had a retrospective observational design, informed consent was obtained a posteriori from surviving patients. For non-survivors, data were included under a waiver of consent, as approved by the institutional review board (Ethics committee “Comitato Etico per le Sperimentazioni Cliniche of the Azienda Provinciale per i Servizi Sanitari di Trento”, Approval number #12,795).

### Data collection

Sepsis and septic shock were defined according to Sepsis-3 criteria and clinical judgment. For each included patient, both the first PCT peak-episode and any subsequent PCT peak-episodes were considered in the analysis. For patients who died during a PCT peak-episode, all available measurements up to the time of death were included in the analysis. At ICU admission, PCT, C-reactive protein (CRP), White blood cells (WBC), and baseline clinical and demographic variables were recorded. In addition, severity scores (SOFA and SAPS II), renal function parameters (including creatinine, urea, and estimated glomerular filtration rate), treatments (including vasoactive support, antibiotic therapy, and renal replacement therapy), and clinical outcomes (including ICU survival and ICU-free days) were collected. Serum PCT and creatinine concentrations were measured every 24 h from ICU admission, with daily follow-up determinations performed at approximately 6 a.m. Urine output was continuously monitored and recorded every two hours throughout the ICU stay. All clinical, laboratory, treatment, and outcome data were extracted from the institutional electronic medical record system (Digistat®, Ascom).

The PCT_peak_ was defined as the highest recorded serum PCT concentration (≥ 2 µg/L) before the onset of a sustained decline [28]. A PCT decline was defined as a reduction of at least 5% from the PCT_peak_ over two or more consecutive days. Following an initial decline, and in comparison to the preceding value, any subsequent increase ≥ 2 µg/L was classified as a new PCT _peak_ [29]. These thresholds were selected to identify a consistent downward trend while minimizing the influence of biological and analytical variability, providing a pragmatic definition applicable to a retrospective dataset.

The PCT_valley_ was defined as the lowest PCT value observed after the initial decline, or the first PCT measurement below 0.5 µg/L [15]. The PCT_decay_ was calculated as the daily relative average reduction in PCT from peak to valley, using the following formula:

$$\mathrm{PCT}_\mathrm{decay}=\frac{\frac{(\mathrm{PCT}_\mathrm{peak}-\mathrm{PCT}_\mathrm{valley})}{\mathrm{PCT}_\mathrm{peak}}}{\text{number of days} (\text{peak to valley})}\times 100$$

For each patient, every PCT rise (from its onset to the corresponding PCT _peak_) and the subsequent decline (from PCT _peak_ to PCT _valley_) were treated as independent events in the analysis and defined as PCT peak-episodes. This approach ensured a clear distinction between episode-level and patient-level data. The unit of analysis was then the PCT peak episode, with multiple episodes potentially occurring within the same patient. To appropriately account for the non-independence of observations, linear mixed-effects models were employed, including a random intercept for patient ID. This approach allowed us to explicitly model within-patient correlation and adequately address patient-level clustering.

Missing laboratory values were managed by excluding the corresponding time point from decay calculations, while mortality during the ICU stay did not prevent the inclusion of available PCT_peak_–episodes up to the time of death.

Patients were stratified according to the AKI-KDIGO classification [30]. The estimated glomerular filtration rate (eGFR) was calculated daily using the Cockcroft-Gault formula, based on each serum creatinine value collected during the ICU stay. AKI classification was based on the ratio of serum creatinine to the creatinine nadir during ICU stay, together with urine output criteria (see Supplementary Table S1).

Each PCT peak-episode was classified in KDIGO stage based on creatinine and urinary output values from the corresponding PCT_peak_ day. This was an adaptation of standard KDIGO criteria, intentionally applied to align renal function assessment with the specific time point of PCT measurement and to enable a time-matched analysis of PCT kinetics. Episodes were then assigned to the appropriate renal severity group for each episode, enabling comparative analysis of PCT peak, valley, and decay.

RRT was initiated based on standard clinical indications, including refractory fluid overload, severe electrolyte or acid–base disturbances, uremic complications, and persistent oliguria or anuria. In our ICU, RRT was delivered as continuous veno-venous hemodialysis or hemodiafiltration using a standardized filter, with routine replacement every 72 h or earlier if needed. Anticoagulation strategies were applied according to clinical judgment.

### Statistical analysis

All analyses were performed using R® software Version 4.3.2. Continuous variables are reported as mean ± standard deviation when normally distributed, or as median and interquartile range (IQR) when non-normally distributed. The Kolmogorov–Smirnov test was used to assess the normality of data distribution. Comparisons between groups were performed using the Kruskal–Wallis test for non-parametric data and ANOVA for parametric data. When applicable, Bonferroni post hoc tests were applied to identify statistically significant differences between groups. To account for the hierarchical structure of the data and the non-independence of repeated observations, linear mixed-effects models were applied, including a random intercept for patient ID. A *p*-value < 0.05 was considered statistically significant.

## Results

**Fig. 1:**
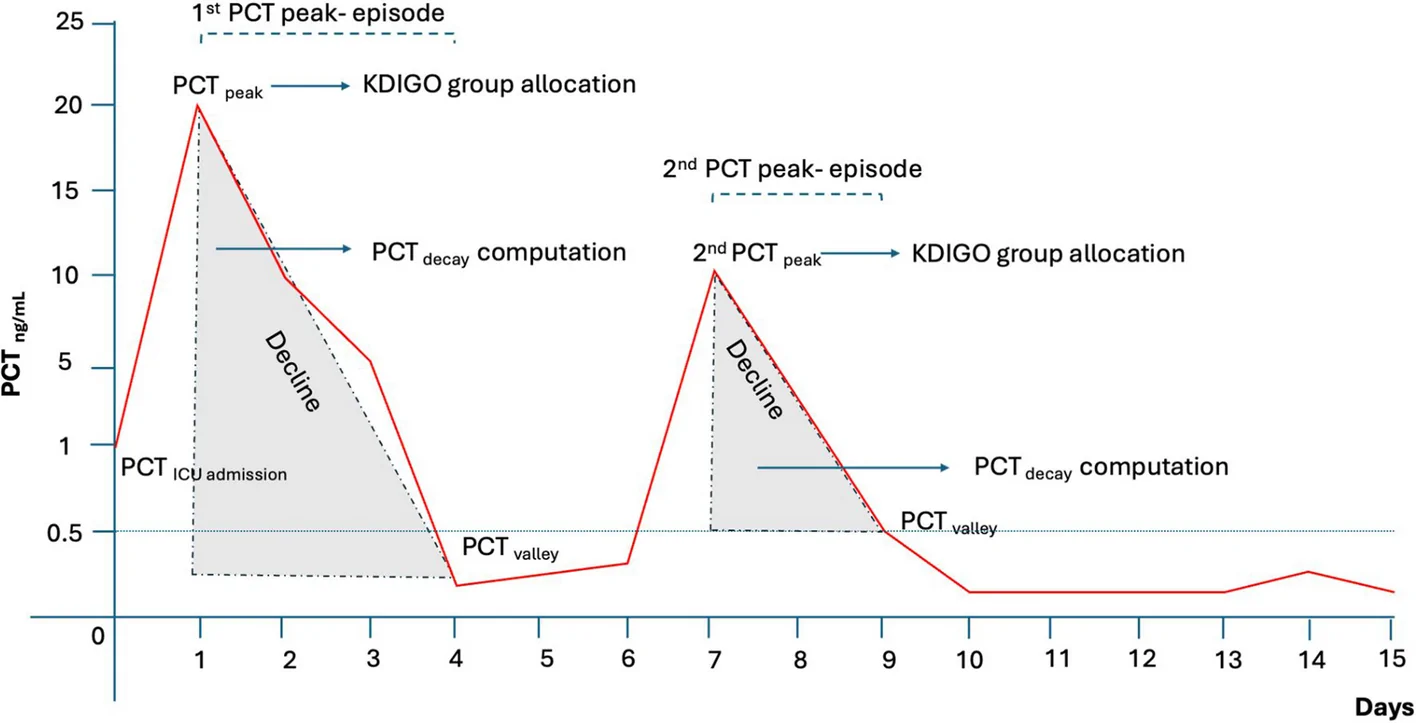
Graphical representation of the study variables

During the study period 405 patients were admitted to the ICU; 9 were excluded due to consent denial or missing data in key variables required for the analysis, specifically including PCT measurements, daily serum creatinine values, and urine output recordings, resulting in a final study population of 396 patients (see CONSORT flow chart in Supplementary Fig. S1). At ICU admission, 82 patients (20.7%) were classified in the no-AKI group, 176 patients (44.4%) in stage 1, 65 patients (16.4%) in stage 2, and 73 patients (18.4%) in stage 3. Among patients in stage 3, 12.3% underwent RRT (see Table 1). No significant differences were observed in the incidence of sepsis/septic shock at ICU admission across the four KDIGO groups. Similarly, there were no statistically significant differences in SOFA and SAPS II scores, vasopressor use, or antibiotic administration (Table 1). Twenty-eight-ICU-free days and survival rates were significantly lower in patients with severe acute kidney injury (Table 1; *p* < 0.001) (Fig. 1).

**Table 1:** Baseline characteristics and ICU outcomes of the study population

		KDIGO groups				
		No AKI	Stage 1	Stage 2	Stage 3	*p* value
Number		82	176	65	73	
Age		64 [47, 74]	67 [55, 77]	65 [53, 74]	73 [63, 78]	0.029
BMI		24 [21, 26]	25 [23, 27]	26 [22, 29]	26 [22, 31]	0.001
Gender (%)	Female	27 (32.9)	50 (28.6)	25 (38.5)	35 (47.9)	0.387
Sepsis/septic shock (%)		34 (41.5)	71 (40.3)	32 (49.2)	44 (60.3)	0.374
Vasoactive drugs (%)	Use of	38 (80.9)	91 (76.5)	34 (70.8)	40 (75.5)	0.721
Empiric antibiotic therapy (%)	Use of	29 (61.7)	79 (66.4)	30 (62.5)	32 (60.4)	0.865
Specific antibiotic therapy (%)	Use of	10 (21.3)	36 (30.3)	15 (31.2)	8 (15.1)	0.130
Prophylactic antibiotic therapy (%)	Use of	7 (14.9)	13 (10.9)	3 (6.2)	9 (17.0)	0.354
SOFA		7 [0, 12]	6 [0, 19]	6 [1, 18]	7 [0, 16]	0.349
SAPSII		37 [6, 69]	33 [6, 87]	32 [11, 81]	32 [0, 72]	0.815
Creatinine (mg/dL)		1.0 [0.7, 1.4]	1.0 [0.8, 1.5]	1.3 [1.0, 1.7]	2.2 [1.3, 3.3]	< 0.001
Urea (mg/dL)		48.5 [33.2, 66.8]	51.0 [40.0, 69.0]	65.0 [43.0, 88.0]	82.5 [57.5, 154.7]	< 0.001
Urinary output (ml/Die)		2430 [1921, 3018]	1510 [1250, 1902]	1390 [970, 1920]	350 [96, 840]	< 0.001
Urinary output (ml/kg/H)		1.31 [1.15, 1.87]	0.81 [0.68, 0.98]	0.74 [0.45, 1.18]	0.14 [0.01, 0.29]	< 0.001
Urinary output (ml/H)		101 [80, 126]	63 [52, 79]	58 [40, 80]	11 [1, 25]	< 0.001
eGFR (ml/min)		84 [58, 147]	85 [52, 133]	76 [54, 139]	54 [28, 77]	< 0.001
Use of RRT (%)		0 (0.0)	0 (0.0)	0 (0.0)	9 (12.3)	< 0.001
ICU Survival (%)		89 (77.4)	120 (75.9)	49 (77.8)	30 (46.2)	< 0.001
28-ICU free days (days)		21 [0, 25]	17 [0, 24]	13 [0, 21]	0 [0, 20]	< 0.001

### Inflammatory markers at ICU admission

**Fig. 2:**
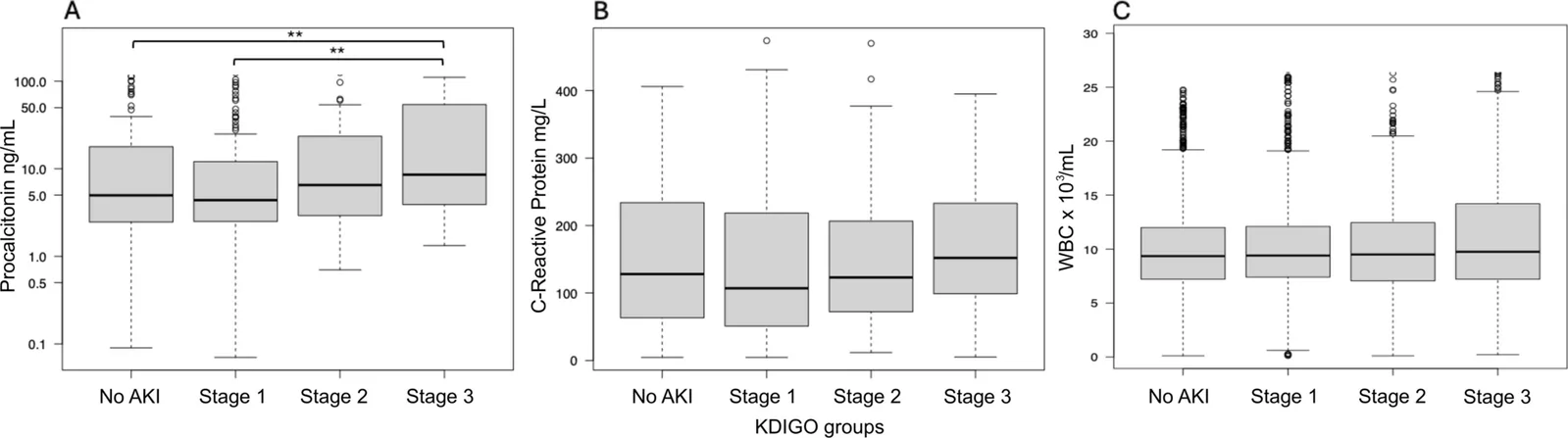
Inflammatory markers at ICU admission stratified by AKI severity (KDIGO classification). **A** (left): median serum PCT levels; **B **(middle): median serum CRP levels; **C **(right): median WBC counts. A significant increase in PCT levels was observed in stage 3 patients compared to no AKI and stage 1 patients. No significant differences were found across the groups for CRP levels and WBC counts

Serum procalcitonin (PCT) levels were significantly higher with increasing severity of renal impairment, from no-AKI and stage 1 groups up to stage 3 (both *p* < 0.01). In contrast, serum C-reactive protein (CRP) levels and white blood cell (WBC) counts did not differ significantly among the groups (respectively *p* = 0.271 and *p* = 0.336) (Fig. 2).

### PCT peak-episodes during ICU stay

The median number of PCT peak-episodes per patient was 1 (IQR 1–1) across all KDIGO groups, indicating a very limited number of repeated observations within the same patient.

**Fig. 3:**
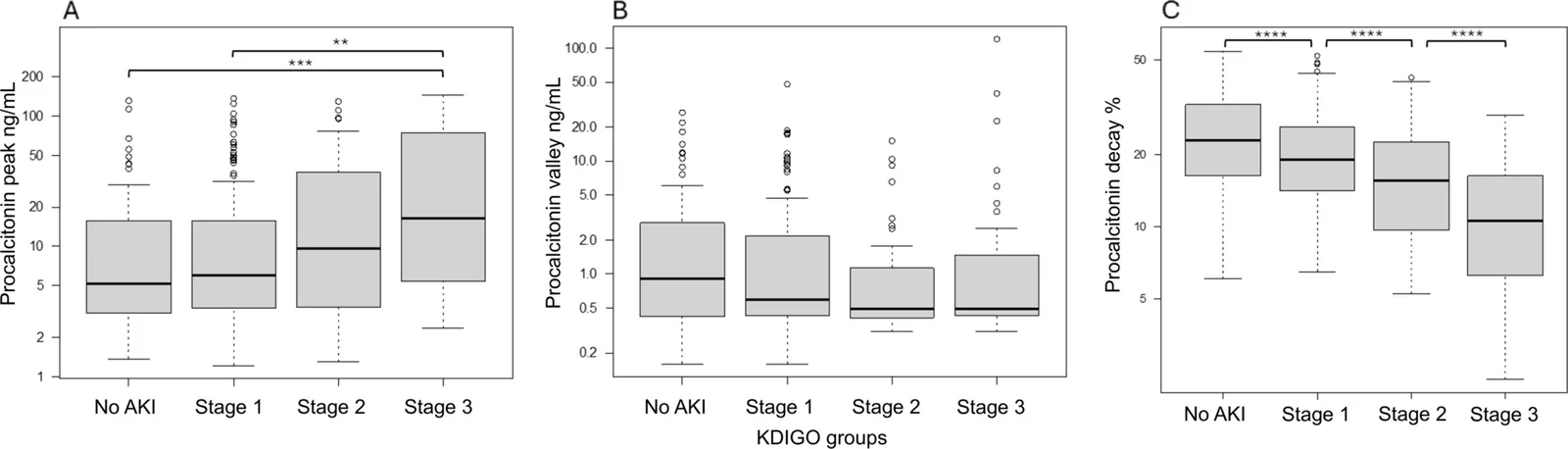
PCT kinetics during ICU stay across AKI stages. **A** (left, *y*-axis) displays the PCT _peak_, **B** (middle, y-axis) shows the PCT_valley_ and **C** (right, *y*-axis) reports the PCT_decay_. As shown, the PCT _peak_ was significantly higher in stage 3 patients compared to those with no AKI and stage 1 AKI. No significant differences were found in the PCT_valley_ across groups while the PCT_decay_ differed significantly among all AKI stages

**Fig. 4:**
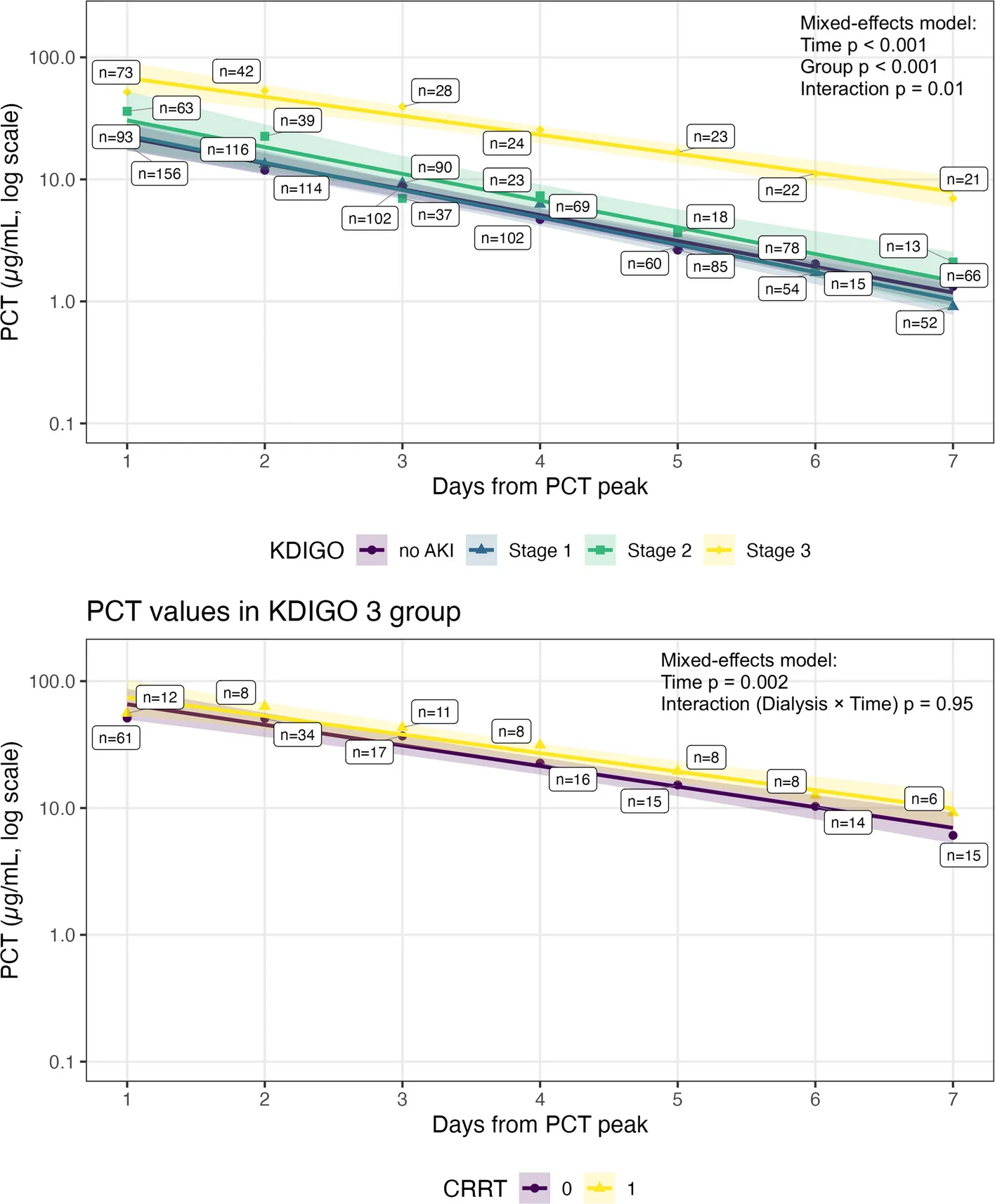
The figure shows model-estimated mean daily PCT levels (y-axis) over the 7 days following the PCTpeak (*x*-axis). Shaded areas represent 95% confidence intervals. The upper panel compares all KDIGO groups, highlighting progressively higher PCT levels with increasing AKI severity. The lower panel focuses on KDIGO stage 3 patients, showing higher overall PCT levels but no significant differences between those treated with or without CRRT

The median values of the PCT_peak_ increased progressively from the no-AKI to stage 3 group (*p* = 0.0014). No significant differences were observed in PCT_valley_ values among the groups (*p* = 0.49), whereas the PCT _decay_ decreased progressively with the worsening of AKI severity (*p* < 0.0001) (Fig. 3). Specifically, the median PCT_decay_ values were reduced by 7.5%, 8.2%, and 13.6% in patients with AKI stages 1, 2, and 3, respectively, compared with patients without AKI. Linear mixed-effects models including a random intercept for patient ID showed that most of the variability in PCT_peak_ and PCT_valley_ values was attributable to between-patient differences (ICC 0.77 and 0.99, respectively), whereas PCT_decay_ exhibited a relatively higher within-patient variability (ICC 0.66). However, given the very limited number of repeated observations per patient (median 1, IQR 1–1), the ability of the mixed-effects model to reliably estimate intra-patient variability is limited. Finally, no statistically significant differences were observed in the daily serum PCT levels between patients undergoing RRT and those with the same AKI stage who did not receive RRT (Fig. 4).

## Discussion

In this study, we describe PCT kinetics in ICU patients stratified by AKI severity and during RRT. Our results can be summarized as follows: (1) at admission, serum PCT levels increased in parallel with the worsening of renal dysfunction, whereas serum CRP levels and WBC counts showed no significant differences; (2) during the ICU stay, PCT_peak_ rose with AKI stage, PCT _valley_ values remained similar, PCT _decay_ declined progressively; and (3) RRT did not significantly affect serum PCT levels.

At ICU admission, patients stratified into four groups based on the severity of acute kidney injury (AKI) exhibited comparable clinical profiles in terms of sepsis/septic shock incidence, illness severity (as assessed by SOFA and SAPS II scores), and the use of vasopressors and antibiotic therapies. Within these cohorts, serum procalcitonin (PCT) levels were significantly higher in patients with mild to severe AKI. Interestingly, other inflammatory markers commonly used in clinical practice, such as white blood cells (WBC) counts and C-reactive protein (CRP), did not show significant differences across the same groups. This finding may indicate a potential association between serum PCT levels and the degree of renal dysfunction, which appears to be at least partly independent of systemic inflammation or infection burden. Notably, our results challenge some previously published data. For instance, Meisner et al. (2000) evaluated PCT clearance in 69 ICU patients with varying degrees of AKI and reported no significant relationship between renal function and serum PCT concentration or clearance [31]. However, their analysis was limited to a 48-h window post PCT_peak_. In contrast, our study was designed with a larger sample size and a prolonged observational period encompassing the entire ICU stay, enabling us to capture PCT kinetics with greater granularity and temporal resolution. These methodological differences may account for the divergent findings and suggest that longer-term monitoring is essential to fully understand PCT behavior in the context of renal dysfunction.

A second key finding emerged from our longitudinal-analysis of PCT kinetics during ICU stay. Patients with AKI exhibited not only higher peaks of serum PCT levels but also a reduced rate of daily PCT decline. Interestingly, the nadir or “valley” values of PCT did not significantly differ across groups, indicating that the delayed decline rather than baseline production may be primarily responsible for elevated PCT in AKI. This is partially at odds with the findings of Meisner et al. (2001), who, in a prospective study involving 67 patients with normal to severe renal dysfunction, assessed both plasma and urinary PCT concentrations over a 48-h period. Although they demonstrated that PCT is at least partially eliminated via the urinary route, they did not observe a statistically significant difference in serum PCT decline across renal function groups [32]. While we did not measure urinary PCT levels, our prolonged, daily plasma monitoring in a larger cohort revealed a significant attenuation in PCT_decay_ among patients with impaired renal function—suggesting that renal clearance, though partial, plays a more critical role over time than previously assumed.

Finally, we evaluated the potential influence of RRT on PCT plasma levels among patients with advanced AKI. Interestingly, we found no significant differences in the daily serum PCT concentrations between patients undergoing RRT and those managed conservatively, despite similar degrees of renal dysfunction. Previous studies have hypothesized that artificial membranes used in RRT may contribute to PCT removal through mechanisms such as ultrafiltration and membrane adsorption [33, 34]. For instance, Nishikura et al. demonstrated that CVVHD could facilitate PCT elimination, while Level et al. reported similar findings during CVVH in patients with septic shock [35, 36]. However, these studies did not determine whether such removal translated into clinically relevant reductions in serum PCT concentrations. Our data suggest that, despite potential membrane-related PCT elimination, the net effect of RRT on circulating PCT levels is negligible—at least within the parameters of the techniques used in our cohort. Taken together, our findings indicate a possible association between serum PCT levels and the degree of renal dysfunction, which appears to persist even after accounting for systemic illness severity or infection markers. In addition, our results suggest that the decline in PCT may be progressively impaired with worsening renal function. Moreover, RRT does not appear to significantly alter PCT levels over time, underscoring the potential utility of PCT as a reliable biomarker in AKI patients regardless of RRT status. These insights could have important implications for the interpretation of PCT values in critically ill patients and warrant further investigation in prospective, mechanistic studies.

## Limitations

This observational study had several limitations. Firstly, baseline serum creatinine values prior to ICU admission were not available for most patients. Therefore, patient classification into AKI KDIGO stages was based on the lowest creatinine value (nadir) recorded during the ICU stay. Additionally, urinary output used for group allocation was calculated as the mean over 24-h periods. Due to the retrospective nature of the study and the presence of missing microbiological data, it was not possible to correlate PCT peak-episodes with microbiological cultures positivity or pathogen identification. However, it should be emphasized that the primary aim of the study was to analyze PCT kinetics independently of the underlying cause of its variation. The definition of PCT decline was based on a ≥ 5% decrease over two consecutive days, a threshold that was pragmatically selected and not externally validated. Although this approach was intended to reduce the impact of biological and analytical variability, its arbitrary nature may limit comparability with other studies and influence the interpretation of PCT kinetics.

Multiple PCT peak-episodes may occur within the same patient. Although mixed-effects models were applied to account for within-patient clustering, the retrospective design limits the ability to fully control for all potential confounders at the individual level. Consequently, repeated episodes in the same patient may reflect heterogeneous clinical trajectories that cannot be completely disentangled in retrospective analyses. Because PCT kinetics were assessed longitudinally, patients who died during ICU stay contributed fewer observations than survivors, introducing potential informative censoring. Although mixed-effects models may partially mitigate this issue, bias due to differential follow-up cannot be excluded, particularly when interpreting PCT_decay_ in more severe cases. Furthermore, within our ICU population, only two renal replacement therapy (RRT) modalities were employed (CVVHD and CVVHDF) using a single filter type (AV1000). The delivered dialysis dose was not recorded and therefore could not be analyzed. The absence of statistically significant differences in the RRT analysis should be interpreted with caution accordingly with the limited sample size of the RRT group. These factors limit the generalizability of our findings and highlight the need for prospective studies with more comprehensive data collection.

## Conclusion

Despite some limitations, our study suggests that AKI may increase peak serum PCT concentrations and slow down its decline, while RRT does not seem to have a significant impact. These findings highlight the need to consider renal function when interpreting PCT values in critically ill patients.

## Supplementary Information

Supplementary Material 1: Table S1. AKI KDIGO classification criteria. Figure S1. Consort diagram.

## Data Availability

The datasets generated and/or analysed during the current study are not publicly available due to patient privacy and confidentiality restrictions, but are available from the corresponding author on reasonable request.

## Abbreviations

AKI: Acute kidney injury
ANOVA: Analysis of variance
CRP: C-reactive protein
CRRT: Continuous renal replacement therapy
CVVHD: Continuous veno-venous hemodialysis
CVVHDF: Continuous veno-venous hemodiafiltration
eGFR: Estimated glomerular filtration rate
ICU: Intensive care unit
IQR: Interquartile range
KDIGO: Kidney Disease Improving Global Outcomes
PCT: Procalcitonin
RRT: Renal replacement therapy
SAPS II: Simplified Acute Physiology Score II
SOFA: Sequential Organ Failure Assessment
WBC: White blood cell count
