# Supplementary material for: Procalcitonin kinetics in critically ill patients: a retrospective analysis of the impact of renal dysfunction and renal replacement therapy
Source: J Anesth Analg Crit Care. 2026 Jul 11;6:125. doi: 10.1186/s44158-026-00405-8 (PMC13640271; doi:10.1186/s44158-026-00405-8)
Supplement: Supplementary file 1 [file 44158_2026_405_MOESM1_ESM.docx]

**Electronic Supplemental Material**

**‘Procalcitonin Kinetics in Critically Ill Patients: A Retrospective Analysis of the Impact of Renal Dysfunction and Renal Replacement Therapy’**

**Table 1**

| **AKI KDIGO classification criteria** | | | |
| --- | --- | --- | --- |
|  | **Creatinine ratio**  (to creatinine nadir) | **Urinary output**  (Mean24H - mL/Kg/H) | **Renal replacement therapy**  (use of) |
| **No AKI** | <1.5 | >1 | No |
| **Stage 1** | >1.5 and <2 | >0.5 and <1 | No |
| **Stage 2** | >2 and <3 | <0.5 and >0.3 | No |
| **Stage 3** | >3 | <0.3 | Yes |

**Definition of KDIGO stages for AKI classification applied in the study.** The table reports the criteria used to define AKI stages according to KDIGO classification, based on serum creatinine ratio relative to the creatinine nadir during ICU stay, urine output thresholds, and the use of renal replacement therapy (RRT). These criteria were applied to classify each PCT peak-episode into the corresponding renal severity group.

Supplemental figure 1. Consort diagram
